# Extracellular Vesicles for the Treatment of Radiation-Induced Normal Tissue Toxicity in the Lung

**DOI:** 10.3389/fonc.2020.602763

**Published:** 2021-03-02

**Authors:** Pierre Montay-Gruel, Yafeng Zhu, Benoit Petit, Ron Leavitt, Mike Warn, Erich Giedzinski, Jonathan Ollivier, David A. Sinclair, Marie-Catherine Vozenin, Charles L. Limoli

**Affiliations:** ^1^ Department of Radiation Oncology, University of California, Irvine, CA, United States; ^2^ Department of Genetics, Blavatnik Institute, Paul F. Glenn Center for the Biology of Aging Research, Harvard Medical School, Boston, MA, United States; ^3^ Laboratory of Radiation Oncology, Department of Radiation Oncology, Lausanne University Hospital and University of Lausanne, Lausanne, Switzerland

**Keywords:** extracellular vesicles, human embryonic stem cells, proteomics, radiation-injury, lung fibrosis

## Abstract

Human stem cell-derived extracellular vesicles (EV) provide many advantages over cell-based therapies for the treatment of functionally compromised tissue beds and organ sites. Here we sought to determine whether human embryonic stem cell (hESC)-derived EV could resolve in part, the adverse late normal tissue complications associated with exposure of the lung to ionizing radiation. The hESC-derived EV were systemically administered to the mice *via* the retro-orbital sinus to explore the potential therapeutic benefits following exposure to high thoracic doses of radiation (14 Gy). Data demonstrated that hESC-derived EV treatment significantly improved overall survival of the irradiated cohorts (*P* < 0.001). Increased survival was also associated with significant reductions in lung fibrosis as quantified by CBCT imaging (*P* < 0.01, 2 weeks post-irradiation). Qualitative histological analyses revealed reduced indications of radiation induced pulmonary injury in animals treated with EV. EV were then subjected to a rigorous proteomic analysis to ascertain the potential bioactive cargo that may prove beneficial in ameliorating radiation-induced normal tissue toxicities in the lung. Proteomics validated several consensus exosome markers (*e.g.*, CD68) and identified major classes of proteins involved in nuclear pore complexes, epigenetics, cell cycle, growth and proliferation, DNA repair, antioxidant function, and cellular metabolism (TCA cycle and oxidative phosphorylation, OXYPHOS). Interestingly, EV were also found to contain mitochondrial components (mtDNA, OXYPHOS protein subunits), which may contribute to the metabolic reprograming and recovery of radiation-injured pulmonary tissue. To evaluate the safety of EV treatments in the context of the radiotherapeutic management of tumors, mice harboring TC1 tumor xenografts were subjected to the same EV treatments shown to forestall lung fibrosis. Data indicated that over the course of one month, no change in the growth of flank tumors between treated and control cohorts was observed. In conclusion, present findings demonstrate that systemic delivery of hESC-derived EV could ameliorate radiation-induced normal tissue complications in the lung, through a variety of potential mechanisms based on EV cargo analysis.

## Introduction

Compared to stem cell therapies, the ability of extracellular vesicles (EV) to stimulate regenerative healing while eliminating risks of teratoma/tumor formation and confounding complications associated with immune suppression, indicate their potential translational utility. While regenerative approaches for implementing stem cell treatments in the context of radiation injury hold tremendous potential (1), EV circumvent certain stem cell-based caveats due to their low immunogenicity, long circulating half-life, and ability to cross the blood-brain barrier (2–4). Recent work from our laboratory has demonstrated the functional equivalence of EV and human stem cells in resolving radiation-induced cognitive dysfunction and associated pathology following intra-cranial delivery to the irradiated hippocampus (5, 6).

EV are secreted by nearly every mammalian cell type and contain a wealth of bioactive cargo capable of modulating target cell physiology and function though a variety of paracrine signaling mechanisms (7). Depending on such factors as cellular origin, cargo contents, membrane composition, and target cell indications, interactions of EV with damaged, diseased or otherwise compromised tissue beds can promote functional recovery (3, 7). As membrane bound vesicles, EV are typically divided into two groups based on size and mode of formation. Microvesicles (MV) tend to be larger (100 nm–1 µm) and are directly assembled from cellular contents and released by outward budding of the cell membrane (8). Exosomes are smaller (30–100 nm) intraluminal vesicles within endosome-derived multivesicular bodies (MVB) that then fuse with and release from the plasma membrane (9). For the resolution of radiation injury, no clear evidence has demonstrated a therapeutic advantage of MV over exosomes or *vice versa*, although there are many distinctions between these different subclasses of EV (10). For this reason, EV-based treatments included the full-size range of vesicles secreted into the conditioned medium.

Migration of EV through the extracellular space or circulation provides the routes whereby EV can interact with target cells, presumably through interactions between transmembrane proteins on the EV and specific receptors on the surface of the target cell. Recipient cells internalize EV *via* either fusion with the plasma membrane or more commonly by endocytosis (11). This then initiates the functional transfer of critical bioactive cargo containing lipids, proteins, organelles, and an assortment of nucleic acids including microRNA (miRNA). The ability of EV to target and functionally interact within the radiation-injured tissue bed provides a heretofore unexplored area for resolving a wide range of dose limiting normal tissue toxicities associated with radiation exposure including the radiotherapeutic management of cancer. Here, through a series of proof of principle studies, we highlight the remarkable abilities of human embryonic stem cell (hESC)-derived EV, delivered systemically to functionally resolve radiation-induced lung toxicity, and importantly, without promoting tumor growth. Furthermore, proteomic analysis of EV contents identified a variety of potentially beneficial protein classes including those comprising complexes I–V of the mitochondrial electron transport chain. This together with the presence of mitochondrial organelles, points to multiple candidates by which the bioactive EV cargo might functionally resolve radiation-induced normal tissue injury.

## Material and Methods

### Stem Cell Culture and Isolation of EV

Growth, culturing, and maintenance of human embryonic stem cells was approved by the Institutional Human Stem Cell Research Oversight (HSCRO, #2007-5629) and Institutional Biosafety (IBC) Committees. The hESC line H9 (WA09 Wicell Research Institute, Inc., Madison, WI) was cultured and expanded in Nutristem XF medium (Biological Industries, Cat# 05-100-1A; Cromwell, CT) in a humidified incubator (5% CO_2_, 37°C). No exogenous serum was used under any of our culture conditions. Six well tissue culture plates (Corning, NY) were coated with Vitronectin XF diluted in Cell Adhere Dilution Buffer (STEMCELL Technologies, Cat. # 07180; Vancouver, CA). Cells were passaged every 4–6 days with manual selective passaging technique using an EVOS4 microscope. Conditioned medium was collected from cells between passage 45 and 60. Cell pluripotency was confirmed by staining for Oct3/4 and Nanog markers. The cells were shown regularly to test negative for mycoplasma with MycoAlert Mycoplasma Detection Kit (Lonza, Cat# LT07-118; Basel, Switzerland).

For the harvest of conditioned media from hESC, culture medium was changed every day with 2 ml from plating to 50% confluence. At 50% confluence, the medium is replaced with 4 ml per well and conditioned medium harvested from optimal colonies (<5% differentiation) for three days until 80% confluency was achieved. Yield from a single 6 well plate is 48 ml and cell surface area of colonies in one 6 well plate at 50 and 80% confluence is 28.5 cm^2^ and 45.6 cm^2^, respectively. Conditioned medium is briefly stored at 4°C until ~420 ml total volume is obtained, sufficient for most applications described herein.

For the isolation of EV, pooled stocks of conditioned media collected over the duration cell culturing were stored at 4°C before biweekly ultracentrifugation. Details describing EV isolation *via* ultracentrifugation have been described (6). Briefly, while maintaining sterility, conditioned media is spun at 2500×g at 4°C for 20 min to remove subcellular debris and the supernatant is bulk filtered (0.45 µm). The filtrate is transferred to 70 ml polycarbonate ultracentrifuge bottles (Beckman) and spun at 100,000×g at 4°C for 90 min. The supernatant is discarded and pelleted EV are collected in PBS. EV from six isolations are typically pooled into smaller polycarbonate ultracentrifuge bottles for ease of collection and washed with PBS, pelleted once more at 100,000×g at 4°C for 120 min. Concentrated EV are resuspended in small volumes of PBS, quantified and characterized using a Zetaview instrument (ZetaView PMX 110; Meerbusch, Germany) with yields varying between 1×10^9-12^/ml depending on initial conditioned media volumes and pellet recovery efficiency during isolation with a typical size distribution of 100 ± 55 nm (diameter).

### Thoracic Irradiation and Post-Irradiation Survival After hESC-Derived EV Therapy

Animal experiments were approved by the Swiss (VD3236) Ethics Committee for Animal Experimentation and performed within institutional guidelines. Female C57BL/6J mice were purchased from Charles River Laboratories (France) at the age of 10–12 weeks. Mice were anesthetized (2% isoflurane) and received local thoracic irradiation using using a XRad 225Cx irradiator (Precision X-ray). The prescribed dose was determined at 10 mm depth with a 15 mm circular collimated field according to previous depth dose measurements in a solid water phantom. Irradiations were performed at 225 kV, 13 mA, with a 0.3 mm copper filter and delivered after fluoroscan imaging to position the mice at the treatment isocenter. Whole thorax irradiation was performed with two opposite vertical beams delivering 14.4 Gy in total (n = 18 mice). Twenty-four hours post-RT mice were randomly divided in two groups. Control group (n = 7) received iv. injection of PBS whereas the EV group (n = 11) was injected with 1×10^10^ hESC-derived EV. Intravenous injections were performed under isoflurane anesthesia and *via* the retro-orbital sinus.

### Cone Beam Computed Tomography (CBCT) Analysis of Lung Fibrosis

Lung density was monitored at 0, 2, 6, and 12-weeks post-RT by CBCT imaging (80 kV; 1 mA) using the XRad 225Cx system (Pxi Precision X-Ray) and under isoflurane anaesthesia. Lung contouring and reconstruction were performed using the Osirix Lite Software. Lung density was evaluated for each animal and at each time-point by Hounsfield Unit (HU) measurements. Values of ΔHU were calculated for each animal at each timepoint by the formula ΔHU_t(x)_ = HU_t0_ – HU_t(x)_ over the time-course of the experiment at 2, 6, and 12-week post-irradiation. Results are expressed as the ratio between the value obtained at a dedicated time point and the initial base-line value (ΔHU).

### Histological Staining of Lung Fibrosis

Animals were euthanized by cervical dislocation at the first appearance of macroscopic symptoms that included weight loss and respiratory distress syndrome. Lungs were sampled and gently inflated by the injection of 1 ml of FineFIX (#84-1717-00/Biosystems) directly in the trachea. The organs were then fixed in the same solution and kept at 4°C before being paraffin embedded and cut into 4 µm sections. The sections were stained with a solution of hematoxylin-eosin (HE) and Sirius Red, examined using an inverted brightfield microscope (Evos XL Core/Thermo Fisher Scientific).

### Proteomic Analysis of hESC-Derived EV

Lysis buffer (4% SDS, 50 mM HEPES pH 7.6, 1 mM DTT) was added into 80 µl hESC EV sample (in PBS buffer) to reach 200 µl. The total protein amount was estimated (Bio-Rad DC). Protein digestion (LysC and trypsin, sequencing grade modified, Pierce) was performed using a modified protocol for SP3 protein clean up (12) followed by SCX peptide clean up. Each sample was separated using a Thermo Scientific Dionex nano LC-system in a 3 h 5–40% ACN gradient coupled to Thermo Scientific High Field QExactive. The software Proteome Discoverer *vs*. 1.4 including Sequest-Percolator for improved identification was used to search the Homo sapiens Uniprot database for protein identification, limited to a false discovery rate of 1%. We used a precursor ion mass tolerance of 10 ppm, and product ion mass tolerances of 0.02 Da for HCD-FTMS. The algorithm considered tryptic peptides with maximum two missed cleavage; carbamidomethylation (C) as fixed modifications; oxidation (M) as variable modifications. The protein network was generated using Cytoscape v 3.7.2 (13).

### Mitochondrial Assessments

To determine whether hESC-derived EV contained mitochondrial (mt) DNA, PCR was undertaken. Primers designed to amplify across the mt encoded tRNA-Leu(UUR) gene were used under standard amplification conditions (35 cycles: 5 min 95°C; 30 s 62°C; 1 min 72°C). Primers for the forward sequence 5’-CACCCAAGAACAGGGTTTGT-3’ and reverse sequence 5’-TGGCCATGGGTATGTTGTTA-3 yield a 107 bp amplicon unique to the human mt-genome.

### Tumor Studies

Lung carcinoma cells TC1 (14) were injected to generate subcutaneously growing lung tumors in C57Bl6 mice. Cells were cultured in DMEM + 10% FBS + 1% HEPES at 37°C, 5% CO_2_. Fifty thousand cells were injected in 100 μl of PBS solution in the left flank of C57Bl/6J mice under isoflurane anesthesia. The same day, animals were treated with either a sham injection of PBS (Vehicle group, n = 14) or 1x10^10^ hESC-derived EV (EV group, n = 16) injected *via* retro-orbital sinus injection. Tumor growth was evaluated three times a week by caliper measurement and tumor volume was calculated with the hemi-ellipsoid volume formula as: $Volume=\frac{width^{2}\times length}{2}$.

### Statistics

Statistical analyses were carried out using GraphPad Prism (v6) software. CBCT data were analyzed using one-way ANOVA followed by Tukey’s range test. Survival data were analysed using the log-rank test. Tumor growth data were analyzed using Kruskal-Wallis one-way ANOVA followed by Mann-Whitney U test. Data in the text are presented as means ± SD or SEM, and all analyses considered a value of *P* ≤0.05 to be statistically significant.

## Results

### Growth, Isolation, Characterization of hESC-Derived EV

**Figure 1:**
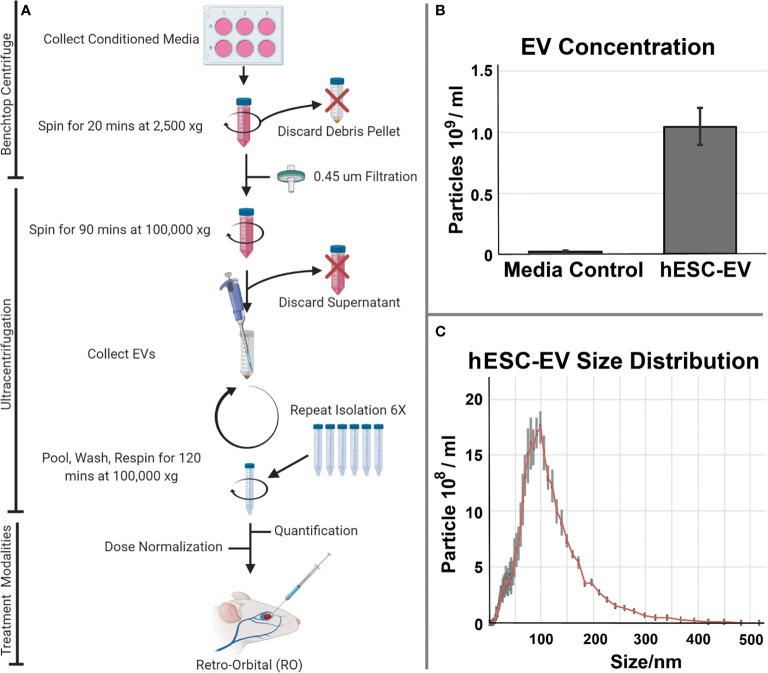
Isolation and characterization of hESC-derived EV isolation and administration. **(A)** Conditioned medium collected from cultures of hESC or hNSC) were processed by ultracentrifugation for the isolation and quantification of EV. EV were subsequently delivered as systemic, retro-orbital (RO) injections to evaluate their potential therapeutic efficacy in resolving radiation-induced injury to the lung. EV isolated from the serum alone or those from actual hESC cultures were subjected to the isolation and purification protocol described. **(B)** Media accounted for a trace fraction (< 4%) of the total hESC-derived EV used in these studies. Mean EV concentration ± SD. **(C)** Size distribution of hESC-derived EV reveals a prominent exosome peak at 100 nm.

Preparations of hESC-derived EV were used to investigate possible benefits of this preclinical strategy to ameliorate radiation-induced normal tissue injury. For these studies we focused on the lung, a target organ known to respond late (many months) after exposure and to express multifaceted injury responses following irradiation. To evaluate the feasibility and therapeutic efficacy of EV delivered systemically, hESC-derived EV were delivered retro-orbitally (RO). Follow up studies then quantified functional outcomes in the lung. A schematic of this approach is presented (**Figure 1A**).

For the hESC-derived EV used in this study, we characterized the background contribution of EV derived from the trace serum in unconditioned culture medium alone versus conditioned medium derived from the hESC cultures. Data shown indicates that the overall contribution of EV from the media serum is negligible (<4%**)** when compared to that obtained in the presence of the cultured hESC (**Figure 1B**). Further size characterization of the hESC-derived EV shows a prominent peak at 100 nm, indicating that the majority of the EV used in the current study were in fact exosomes (**Figure 1C**).

### EV Therapy Rescues Mice From Acute and Delayed Radiation-Induced Lung Injury

**Figure 2:**
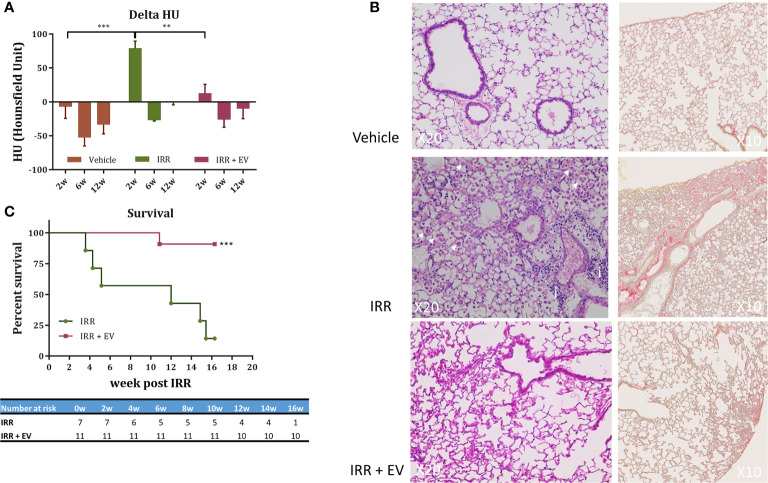
EV therapy rescues mice from acute and delayed radiation injury. **(A)** Cone beam computed tomography (CBCT) was used to longitudinally investigate the occurrence of radiation-induced pulmonary density. Mice (n = 15) were imaged before irradiation to define a base-line level of lung density (HU) for each mouse. This measurement was used to normalize data over the time-course of the experiment. Mice were then divided in three groups: non-irradiated (n = 6), 14.4 Gy irradiated (n = 4), and 14.4 Gy irradiated + EV-treated mice (n = 5) and imaged at 2, 6, and 12-week post-irradiation. Results are expressed as the ratio between the value obtained at a dedicated time point and the initial base-line value (delta HU) ± SEM. Data were analysed using one-way ANOVA and Tukey test ***p < 0.0005 and **p < 0.009. **(B)** Histological staining (HE and Sirius Red staining) showed typical late radiation-induced fibrosis remodeling 16 weeks post-RT. In controls, no signs of radiation injury were evident. However, in the single animal remaining in the IRR group, fibrotic indications included a thickening of alveoli with extracellular matrix and alveolar infiltration with spumous macrophages (arrow) and foci of inflammatory cells (i). In contrast, EV-treated animals showed reduced signs of radiation injury and fibrosis (image/s derived from an animal showing minimal pulmonary fibrosis). **(C)** Survival curves. The percent survival of irradiated (RT) and irradiated plus EV treated (RT+EV) cohorts is shown. Data were analyzed using the log-rank statistical test, ***p < 0.0007.

To extend the applicability of EV-therapies, we investigated their ability to counteract radiation-induced lung injury in mice. A single fibrogenic dose of 14.4 Gy was administered to the whole thorax of mice. Twenty hours post-irradiation EV were RO injected and the occurrence of acute and late lung toxicity was longitudinally followed by cone beam computed tomography (CBCT) over 16 weeks. A significant reduction of pulmonary density was observed at 2 weeks post-irradiation in the EV-treated group as compared with the irradiated group (**Figure 2A**). At later time points, the pulmonary density was similar in all groups (6, 12 weeks post-RT) (**Figure 2A**). The significance of CBCT results at earlier post-irradiation times was explored further at later times using histology which showed preservation of pulmonary structure in EV-treated animals as well as anti-fibrotic efficacy. Sixteen weeks post-irradiation histology indeed showed typical fibrotic remodeling with collagen deposition, macrophage alveolitis and focal inflammatory foci in the irradiated animal, whereas EV-treated animals showed significant reductions in collagen, macrophage infiltration and inflammation (**Figure 2B**). Finally, in irradiated animals, radiation-induced lung toxicity reduced survival as early as 3.5 weeks post-irradiation and only 25% of mice were still surviving 16 weeks post-RT whereas 91% of mice were alive and free of fibrosis in the EV-treated group (**Figure 2C**).

### Proteomic Analysis of hESC-Derived EV

**Figure 3:**
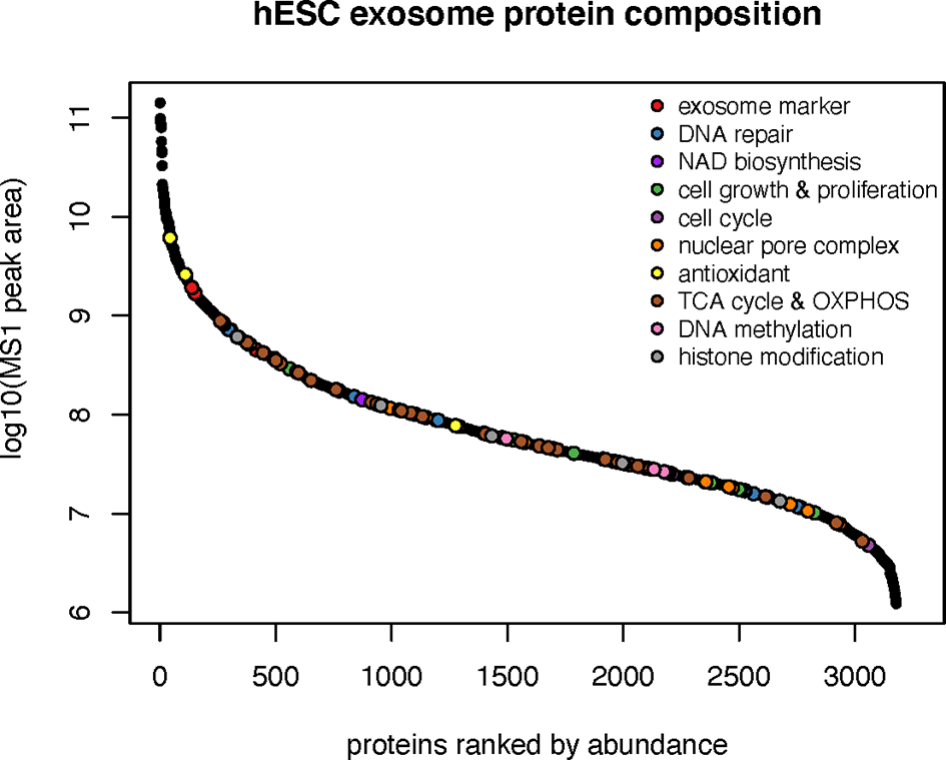
Protein classes present within hESC-derived EV. Relative abundance of the 10 major classes of proteins identified by proteomic analysis of hESC-derived EV.

To ascertain the nature of the bioactive cargo having the potential capability to resolve radiation-induced injury to the lung, we undertook an extensive proteomic analysis of hESC-derived EV. This analysis revealed a surprising wealth of potential cargo capable of impacting a wide range of physiological processes (**Figure 3**). Given the broad scope of cell cycle, growth and proliferation, antioxidant, DNA repair and metabolic proteins present within these hESC-derived EV, we have focused on those protein classes considered most likely to affect the lung at protracted post-irradiation times. Data regarding the other protein classes can be found in the supplemental information (**SI**) section, where the identities of exosome markers (**Supplementary Figure 1**), histone deacetylases and DNA methylation (**Supplementary Figure 2**), nuclear pore complex (**Supplementary Figure 3**), DNA repair and cell cycle (**Supplementary Figure 4**), and NAD biosynthesis (**Supplementary Figure 5**) proteins are provided.

**Figure 4:**
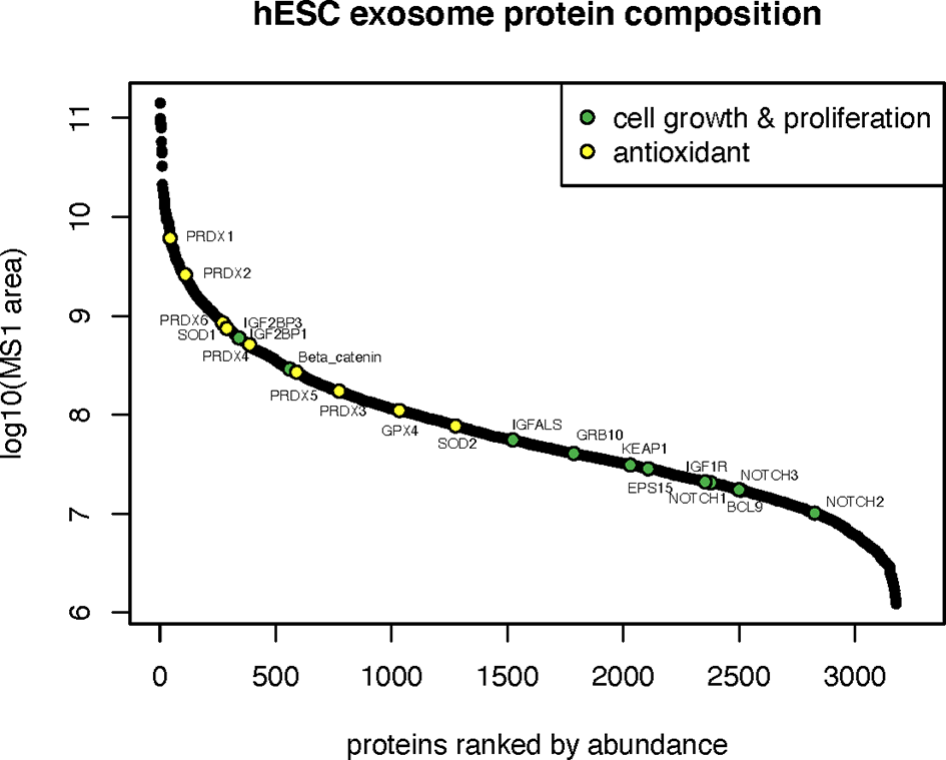
Cell growth, proliferation and antioxidant proteins in hESC-derived EV. Relative abundance of cell growth and proliferation proteins and antioxidant enzymes identified by proteomic analysis.

Proteins involved in cell growth and proliferation influence the recovery of tissues damaged from radiation or compromised by disease and age. Many proteins related to the IGF (IGF2BP1, IGF2BP3, IGFALS, IGF1R, GRB10) and Notch (NOTCH1,2,3) signaling pathways were identified in hESC-derived EV that could clearly impact downstream physiology following irradiation (**Figure 4**). Radiation exposure is known to cause a cascade of oxidative and inflammatory damage that persist in a dose, time and tissue specific manner. In this regard, hESC-derived EV were also found to contain a wealth of antioxidant proteins and in particular all members of the peroxiredoxin family (PRDX1-6) of antioxidant enzymes (**Figure 4**).

**Figure 5:**
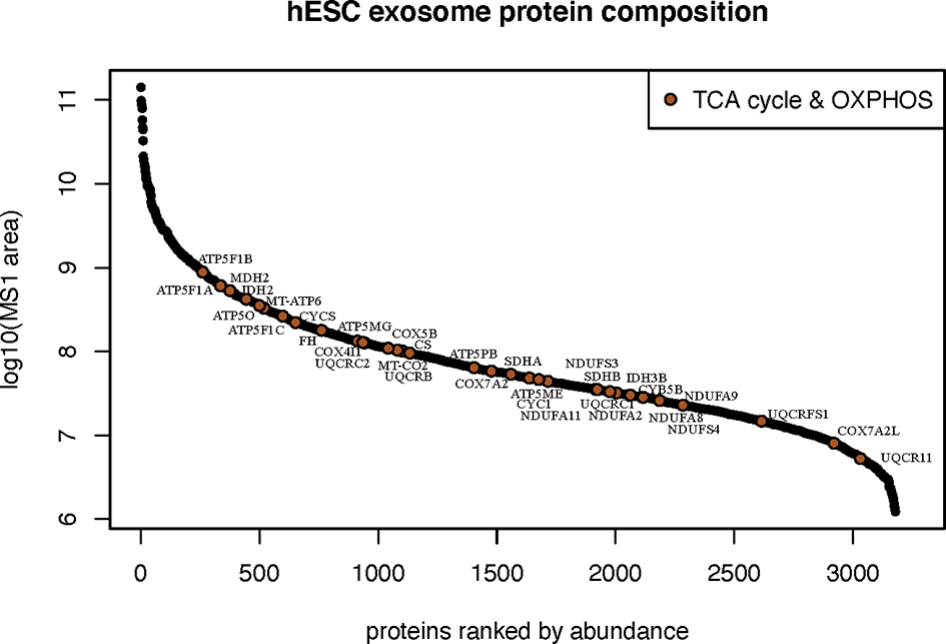
Metabolic bioactive cargo in hESC-derived EV. Relative abundance of metabolic proteins known to participate in the tricarboxylic acid cycle and mitochondrial oxidative phosphorylation identified by proteomic analysis.

The most abundant class of proteins identified were those that mediate cellular metabolism through the tricarboxylic acid (TCA) cycle (or Krebs cycle) and mitochondrial mediated oxidative phosphorylation (OXYPHOS). In all, 34 proteins were identified between these two critical biochemical pathways (**Figure 5**). Proteins and respective subunits important for the TCA cycle include citrate synthase (CS), isocitrate dehydrogenase (IDH2,3B), fumarase (FH), and malate dehydrogenase (MDH2). Proteins involved in mitochondrial OXYPHOS include those of NADH:ubiquinone oxidoreductase (Complex 1: NDUFS3,4; NDUFA2,8,9,11), succinate dehydrogenase (Complex II: SDHA,B), ubiquinol:cytochrome-c oxidoreductase (Complex III: CYCS,CYCS1,CYB5B; UQCRB,C1,C2,FS1,11), cytochrome-oxidase (Complex IV: COX4I1,5B,7A2,7A2L, MT-CO2), and ATP synthase (Complex V: ATP5ME,MG,PB,O; ATP5F1A,B,C; MT-ATP6, MT-CO2). Intriguingly, EV were also found to contain the enzyme nicotinamide phosphoribosyltransferase (NAMPT) that catalyzes the production of nicotinamide mononucleotide (NMN) (**Supplementary Figure 5**).

### Detection of mtDNA Within hESC-Derived EV

The abundance of proteins and enzymatic subunits found in EV that orchestrate the metabolic generation of ATP, including the repertoire of Complex I-V protein subunits mediating oxidative phosphorylation (OXYPHOS) was noteworthy, and prompted efforts to determine whether EV contained other mitochondrial components. The presence of mtDNA in the EV used to resolve radiation-induced lung injury was confirmed by PCR, by the presence of an amplicon specific for the human tRNA(Leu) locus (**Supplementary Figure 6** **).** The presence of mtDNA within EV suggests that certain functional benefits of EV therapy may be derived from the horizontal transfer of mt constituents to target cells during EV fusion events.

### hESC-Derived EV Do Not Enhance the Growth of Xenograft Tumors

**Figure 6:**
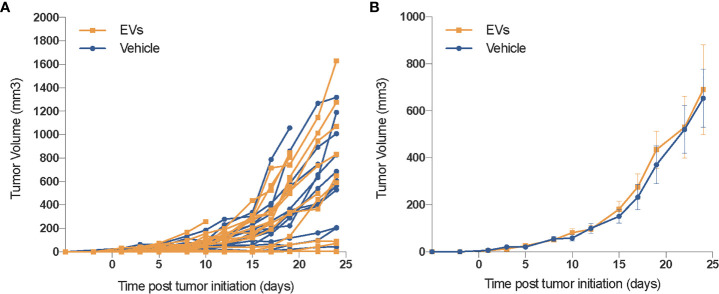
EV do not promote the growth of xenograft tumors. In an initial safety screen, EV were not found to promote the growth of xenograft tumors. Mice injected (SQ) with 50,000 TC1 cells and treated with vehicle or hESC-derived EV the same day, were followed for tumor growth over 1 month. While some individual variation in tumor growth was observed **(A)**, averaged data **(B)** reveals no significant difference in tumor volumes between either cohort. Mean tumor volume ± SEM, *n* = 14–16 animals per group.

While the functional benefits of EV therapy to the irradiated normal lung are promising, in the context of cancer therapy, such benefits must be tempered until their impact on tumor growth can be assessed. To address this important issue, 50,000 TC1 cells were inoculated in the flank of immunocompetent mice and evaluated for changes in growth following identical EV treatments (via RO injections). No difference in tumor growth was observed between the sham-injected and the EV-treated groups, evident from the individual (**Figure 6A**) and grouped (**Figure 6B**) tumor volume measurements. These results show that that hESC-derived EV injected systematically do not influence tumor growth, neither in its initiation nor in the growth rate and independently of other antitumor treatments.

## Discussion

While certain applications of EV-based therapies have begun, their potential for the resolution of radiation-induced normal tissue toxicities remains relatively unexplored. Our past work demonstrating the neuroprotective benefits of cranially grafted EV, when substituted for stem cells, into the irradiated brain laid the foundation for much of the current work. Here we focused on the lung, a late responding organ known to express radiation injury at protracted exposure times. The ability of EV to ameliorate radiation-induced lung fibrosis is noteworthy, especially given that a single treatment (systemic injection) was successful in reducing this serious normal tissue complication. Further, we have demonstrated that a highly beneficial functional outcome could be obtained through a non-surgical route of administration, thereby providing a more tractable and appealing alternative for translating EV therapies to the clinic.

Present data document for the first time, the efficacy of EV-therapy for the resolution of radiation-induced lung injury when administered 24 h post-exposure. Our data suggest that EV-therapy is able to interrupt the acute pathogenic cascade activated at early times after irradiation. In addition, present results show a major reduction of the infiltration of immune cells and macrophages after EV-therapy. The relevance of macrophages is especially interesting in the lung, as recent studies have reported a role for macrophages as critical regulators of fibrosis (15) with an involvement of M2-polarized macrophages in various models of fibrosis including radiation-induced lung fibrosis (16). While the impact of EV-therapy on the activation of major fibrogenic cascades (17) remains to be investigated in detail, the reduction of fibrosis and pulmonary density observed in the present study is consistent with a modulation of proinflammatory and profibrotic signaling pathways in the irradiated lung.

**Figure 7:**
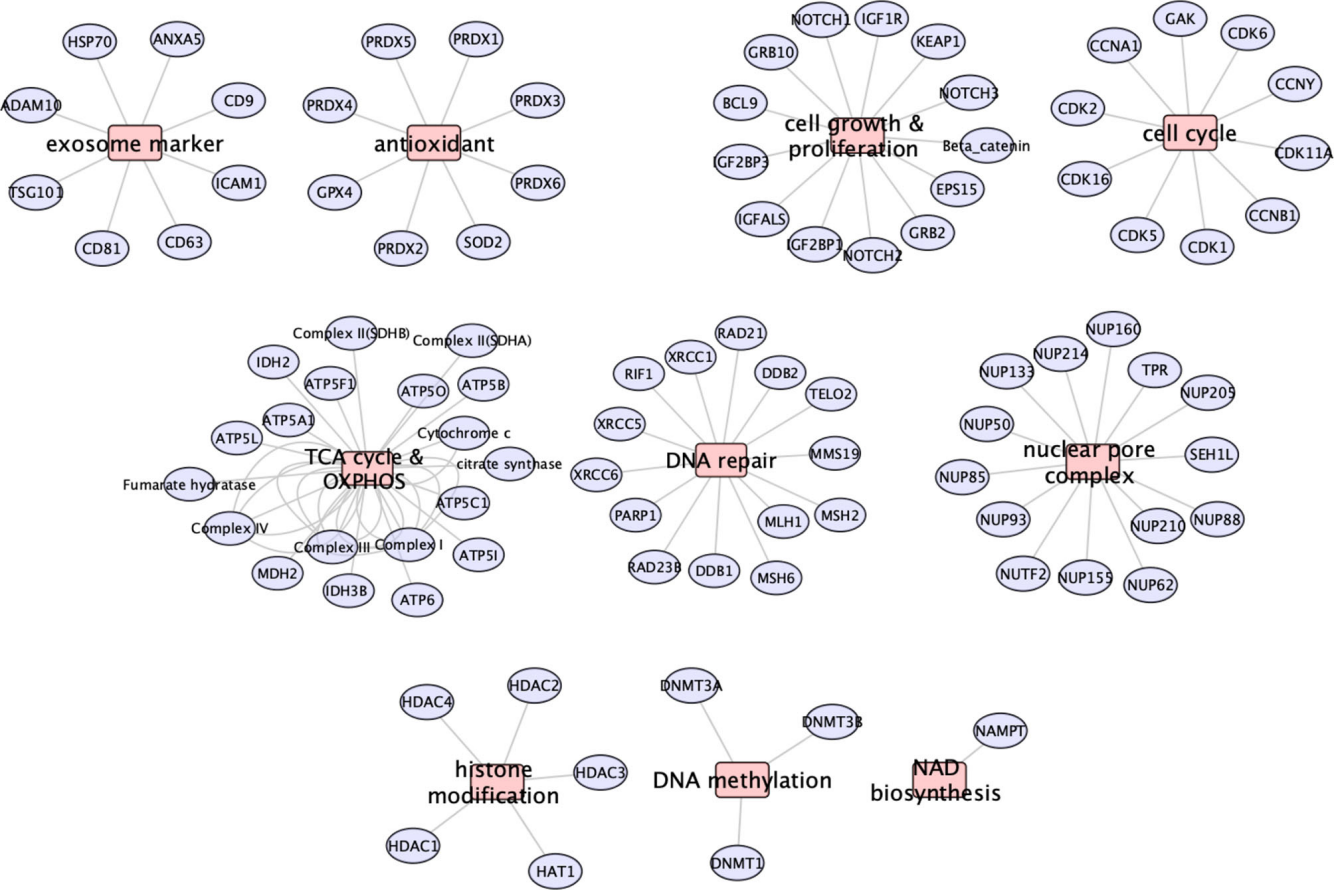
Summary of the major proteins and classes identified in hESC-derived EV by proteomic analysis. Major protein classes show that EV contain exosome markers, antioxidant enzymes, cell growth and proliferation, cell cycle, metabolic (TCA, OXYPHOS, NAMPT), DNA repair, nuclear pore complex and epigenetic modifier (HDAC, DNMT) proteins.

Whether all the beneficial effects of EV on radiation-induced lung pathology are the direct result of the protein (or possibly miRNA) cargo derived through the fusion of EV with alveoli and other pulmonary target cells remains uncertain. Nonetheless, present data indicates the marked capability of hESC-derived EV to resolve a serious complication associated with radiation exposure to the lung, when administered after irradiation. The protein classes identified by proteomic analysis (summarized in **Figure 7**) suggest several potential avenues by which EV fusion events with select target cell of the lung could restore function to radiation injured tissues. By altering the acetylated and methylated landscape of the epigenome, HDAC and DNMT can repress and/or activate multiple gene expression patterns to impact long-term changes in tissue functionality (18). Components of the nuclear pore found in EV could facilitate access and transport of molecules required for nuclear and cytoplasmic repair and homeostatic functions (19).

The presence of proteins able to modulate the signaling and activities of insulin growth factor (IGF) (20–22), NOTCH, Wnt/beta-catenin (23), and KEAP (24), provide multiple routes for modulating cell growth and proliferation in response to radiation and redox stress. Similarly, the presence of various antioxidants such as SOD (25), GPX4 (26) including the PRDX1-6 enzymes (27–32) may provide the capability to recover from pro-oxidant damage produced by prior radiation exposure.

The predominance of TCA enzymes and OXYPHOS proteins identified as EV cargo suggest the potential capability of hESC-derived EV to metabolically reprogram cells to more functionally active states. This idea is supported further by the presence the enzyme NAMPT that catalyzes the production of nicotinamide mononucleotide (NMN). NMN is a co-factor for mitochondrial and sirtuin activities, and has been shown to possess tissue specific-protective roles and to be important in a number of anti-aging pathways (33–35). That metabolic reprogramming may underlie many of the beneficial effects of EV-based therapies is supported by studies in the brain (36, 37), heart (38–41), spinal cord (42), and lung (43–45), where damage from ranging from stroke and ischemia to trauma could in part, be resolved by rejuvenating target cells through mitochondrial replenishment. While multiple strategies involving cellular transfer, injection or transplantation have shown promise [reviewed in (46–49)], the use of EV for similar protective therapies provide a more practical route toward translation.

Augmented ATP production derived from an enhancement of the TCA cycle and OXYPHOS may help maintain, or redirect cellular energetics to optimize macromolecular syntheses necessary for cellular repair and survival. Indeed, the preponderance of electron transfer proteins within EV suggest that such metabolic modulation could be driven by the transfer of mt, mt fragments or other mt components from EV to target cells to rescue and/or augment aerobic respiration (50). Based on the average size of our EV (100 nm) and that of an intact mt (0.5–1 μm) it seems plausible that EV retained mt membrane fragments bound with OXYPHOS proteins in addition to mt DNA. Additional work will be needed to assess whether human mtDNA and/or mt components were in fact transferred to target cells in the murine pulmonary epithelium, evidence that would serve to further substantiate the functional relevance of mt EV cargo.

As the therapeutic benefits of EV are finding broader applicability [reviewed in (3, 7)], in part due to their ease of administration and minimal immunogenicity, certain safety concerns in the context of resolving cancer treatment-induced normal tissue toxicities warrant further consideration. Radiation remains a primary treatment modality for lung cancer and is used in roughly half of all cancer cases. The use of EV to ameliorate radiation-induced toxicities is most logical in the adjuvant setting, where only residual disease might confound this therapeutic approach. Nonetheless, the capability of EV to stimulate secondary tumor regrowth, formation and/or activate distal disease sites is a potential confounder to the use of EV following cancer therapy. While this question will require further and more rigorous evaluation, our proof-of-principle study did provide the first evidence that EV derived from hESC did not promote the growth of implanted tumors over a 1 month follow up. While risky to generalize these findings to other EV and cancer types, data to date does not suggest that stem-cell derived EV would be unsafe under certain clinical settings.

Further investigations are now required to pinpoint the relevant functional roles of different protein classes following radiation injury. Nonetheless, potential target cell interactions with hESC-derived EV afford multiple opportunities to exploit a plethora of bioactive cargo in tailoring a physiological response to prior insult. So where does the field of EV therapy stand for the treatment of radiation-induced normal tissue toxicities? Future studies should seek to define optimal cellular sources of EV to delineate the mechanism of action, to identify bioactive cargo (not exclusive to miRNA) and to pinpoint optimal EV dosing regimens. While current data points to several possible options for EV administration, systemic (RO) or intravenous (in human) routes are likely to provide the best combination of widespread availability and feasibility for repeated treatment regimens. While the lack of teratoma formation and reduced immunogenic response inherent to EV therapies are clear benefits, certain safety issues remain to be thoroughly addressed, especially in the area of cancer treatments. Further work must determine whether such approaches activate “cold” or latent cancers or alter the growth of recurrent malignancies when administered after the cessation of specific cancer treatments. Despite the caveats associated with any burgeoning therapy, EV provide a potentially attractive therapeutic avenue for resolving normal tissue toxicities associated with radiotherapy. Studies here provide the proof of principal that highlight the tremendous potential of EV-based therapy and underscore that such pursuits are clearly warranted.

## Data Availability Statement

The mass spectrometry proteomics data have been deposited to the ProteomeXchange Consortium *via* the PRIDE partner repository with the dataset identifier PXD023213.

## Ethics Statement

The animal study was reviewed and approved by Canton de Vaud Ethics Committee for Animal Experimentation, approval no. VD3236.

## Author Contributions

PM-G and YZ contributed equally. M-CV and CL contributed equally. All authors contributed to the article and approved the submitted version.

## Funding

This work was supported by the Swiss National Science Foundation, Early Postdoc.Mobility No. P2LAP3_187706, the ISREC Foundation thank to a Biltema donation (M-CV), a grant from GuangDong Science and Technology Department No. 2020B1212060018 (YZ) NIH grant DP1AG058605 (DS), and NINDS grant R01 NS074388 (CL).

## Conflict of Interest

DS is a board member of, inventor on patents licensed to, advisor to, and owns equity in EdenRoc and affiliates including Liberty Biosecurity and Life Biosciences and affiliates. Additional disclosures are at https://genetics.med.harvard.edu/sinclair/people/sinclair-other.php.

The remaining authors declare that the research was conducted in the absence of any commercial or financial relationships that could be construed as a potential conflict of interest.
