# Supplementary material for: Extracellular Vesicles for the Treatment of Radiation-Induced Normal Tissue Toxicity in the Lung
Source: Front Oncol. 2021 Mar 2;10:602763. doi: 10.3389/fonc.2020.602763 (PMC7962869; doi:10.3389/fonc.2020.602763)
Supplement: Supplementary file 1 [file DataSheet_1.docx]

**Supplementary Materials**

**Supplemental Figure 1: Exosome markers in hESC-derived EV.** Relative abundance of exosome markers detected by proteomic analysis of the overall population of EV used in this study.

**Supplemental Figure 2: Epigenetic modifiers found in hESC-derived EV.** Relative abundance of histone deacetylases and DNA methyltransferases identified by proteomic analysis.

**Supplemental Figure 3: Nuclear pore complex proteins in hESC-derived EV.** Relative abundance of nuclear pore complex protein subunits identified by proteomic analysis.

**Supplemental Figure 4: DNA repair and cell cycle in hESC-derived EV.** Relative abundance of DNA repair and cell cycle proteins identified by proteomic analysis.


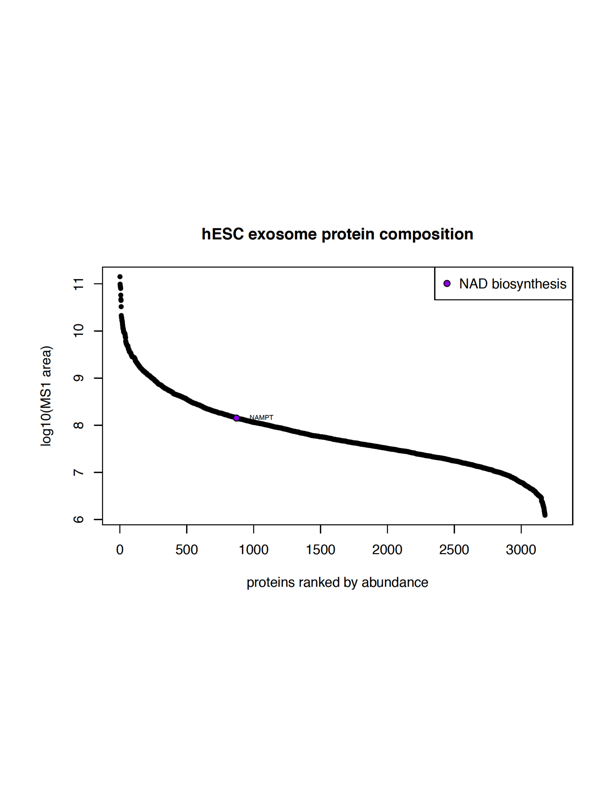


**Supplemental Figure 5: NAD biosynthesis protein in hESC-derived EV.** Relative abundance of NAMPT identified by proteomic analysis.


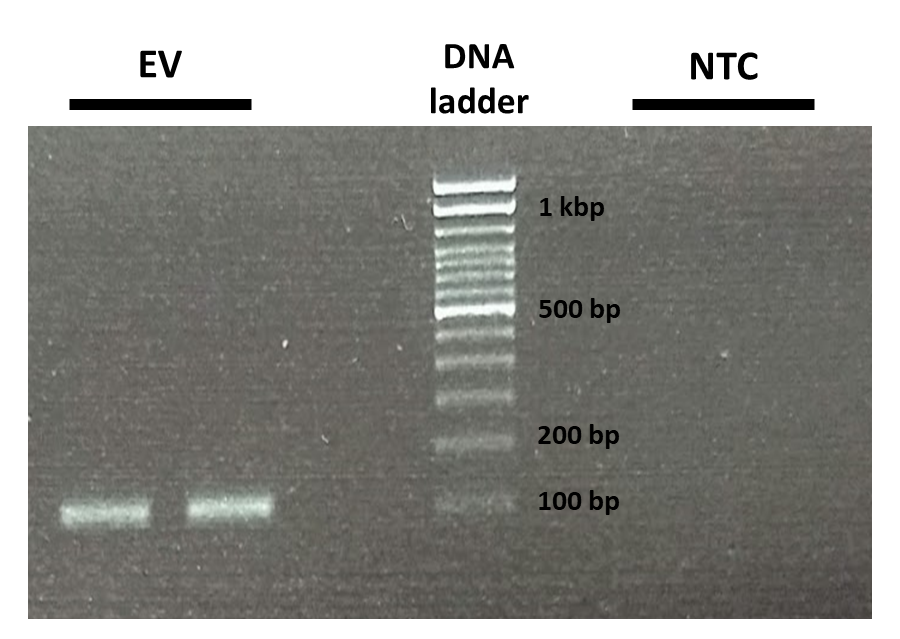


**Supplemental Figure 6: Identification of mtDNA within hESC-derived EV.** EV used to treat radiation-induced lung injury were assessed for the presence of mtDNA by PCR. The presence of the human specific amplicon for the tRNA-Leu(UUR) gene confirmed the presence of mtDNA within the EV. NTC, Non-treated control (lacking EV sample).
